# Significance of the neutrophil-to-lymphocyte ratio in predicting the response to neoadjuvant chemotherapy in extremity osteosarcoma: a multicentre retrospective study

**DOI:** 10.1186/s12885-021-09130-7

**Published:** 2022-01-04

**Authors:** Haijun Tang, Dehuai Liu, Jili Lu, Juliang He, Shuyu Ji, Shijie Liao, Qingjun Wei, Shenglin Lu, Yun Liu

**Affiliations:** 1 Department of Orthopaedics, Minzu Hospital of Guangxi Zhuang Autonomous Region, Nanning, Guangxi China; 2 People’s Hospital of Baise, Baise, Guangxi China; 3 Departments of Surgery of Bone and Soft Tissue Tumor, Affiliated Tumor Hospital of Guangxi Medical University, Nanning, Guangxi China; 4 Departments of General Practice, The First Affiliated Hospital of Guangxi Medical University, Nanning, Guangxi China; 5 Departments of Orthopedics, The First Affiliated Hospital of Guangxi Medical University, Nanning, Guangxi China; 6 Department of Spine and Osteopathic Surgery, The First Affiliated Hospital of Guangxi Medical University, Nanning, Guangxi China

**Keywords:** Neutrophil-to-lymphocyte, Neoadjuvant chemotherapy, Osteosarcoma

## Abstract

**Background:**

At present, no predictive factor has been validated for the early efficacy of neoadjuvant chemotherapy (NACT) in osteosarcoma. The purpose of this study was to investigate the significance of the neutrophil-to-lymphocyte ratio (NLR) in predicting the response to NACT in extremity osteosarcoma.

**Methods:**

Pathological complete response (pCR) was used to assess the efficacy of NACT. Receiver operating characteristic (ROC) curves and the Youden index (sensitivity + specificity-1) were used to determine the optimal cut-off values of the NLR. Univariate and multivariate analyses using logistic regression models were conducted to confirm the independent factors affecting the efficacy of NACT.

**Results:**

The optimal NLR cut-off value was 2.36 (sensitivity, 80.0%; specificity, 71.3%). Univariate analysis revealed that patients with a smaller tumour volume, lower stage, lower NLR and lower PLR were more likely to achieve pCR. Multivariate analyses confirmed that the NLR before treatment was an independent risk factor for pCR. Compared to patients with a high NLR, those with a low NLR showed a more than 2-fold higher likelihood of achieving pCR (OR 2.82, 95% CI 1.36-5.17, *p* = 0.02).

**Conclusion:**

The NLR is a novel and effective predictive factor for the response to NACT in extremity osteosarcoma patients. Patients with a higher NLR showed a lower percentage of pCR after NACT.

## Introduction

Osteosarcoma, which originates from mesenchymal tissue and is mainly located in the long bones, is one of the most common primary malignant tumours in children and adolescents [1]. Before the 1970s, the main treatment method for this disease was amputation and postoperative chemotherapy. However, the prognosis of patients treated with this combination was extremely poor, and the 5-year survival rate was approximately 42% [2]. In 1970, Cortes [3] and Rosen [4] proposed preoperative chemotherapy, which is also known as neoadjuvant chemotherapy (NACT). Since then, the survival rate has increased to 60-70%, and limb salvage is possible for most patients [5].

According to National Comprehensive Cancer Network (NCCN) recommendations, the curative effect of chemotherapy is a key index for assessing whether limb salvage is feasible [6]. Hence, predicting the efficacy of NACT before surgery is very important. Recently, pathological complete response (pCR) has been regarded as the gold standard to estimate the efficacy of NACT [7]. However, pCR is determined based on postoperative tumour specimens and therefore cannot be validated as a predictive factor for the early efficacy of NACT. Additionally, some radiological parameters, including tumour volume, the apparent diffusion coefficient (ADC) on MRI, and standardized uptake values (SUVs) on ^18^F-FDG PET, have also been proposed as predictive indices [8, 9]. Although these parameters can be measured before surgery and have relatively satisfactory reliability, measurement errors, specificity and sensitivity, and costs still need to be improved. Therefore, identifying new, reliable, and inexpensive parameters is important.

The systemic inflammatory response (SIR) has been demonstrated to play a key role in tumorigenesis, metastasis, and even drug resistance [10]. The neutrophil-to-lymphocyte ratio (NLR) and platelet-to-lymphocyte ratio (PLR) are two important indices that reflect the SIR [11] and have also been studied for pCR prediction in various solid tumours, such as breast and oesophageal cancers [12, 13]. Many articles have demonstrated that inflammation-based indicators, such as the NLR, PLR and CRP/Alb ratio, can predict the prognosis of osteosarcoma patients [14, 15]. However, the potential prognostic value of these indicators for pCR after NACT for osteosarcoma has not been reported.

Thus, we conducted this multicentre retrospective study to determine whether the NLR or PLR can be used to predict pCR after NACT in osteosarcoma and therefore serve as a parameter to guide chemotherapy and surgical planning. To the best of our knowledge, this study is the first to explore the association of the response to NACT with the NLR and PLR in osteosarcoma.

## Patients and methods

### Patients

This multicentre study was conducted in three medical institutions. The medical records of patients diagnosed with osteosarcoma confirmed by postoperative pathology from September 2008 to December 2018 were retrospectively reviewed. The inclusion criteria were as follows: (1) patients diagnosed with osteosarcoma by postoperative histopathology; (2) patients who received standard neoadjuvant chemotherapy (NACT) before surgery; (3) patients with complete laboratory data before NACT; (4) patients who underwent tumour resection or amputation such that pathological complete response (pCR) was obtainable; and (5) patients with lesions located in the extremities. The exclusion criteria were as follows: (1) patients with infection, fever, or any blood disease; (2) patients with recurrence; and (3) patients with incomplete medical records.

The clinical and pathological stages of the tumours were defined using the Enneking classification [16]. Informed consent was obtained from all patients, and this study was supported by the Ethical Association of our institution.

### Blood samples and data review

Blood samples were obtained when the patient was hospitalized to start the first cycle of chemotherapy. The NLR was calculated from routine peripheral blood examination results and defined as the absolute number of neutrophils divided by the lymphocyte count. The same formula was applied to determine the platelet-to-lymphocyte ratio (PLR). Other parameters, including white blood cell count (WBC), percentage of monocytes, erythrocyte sedimentation rate (ESR), C-reactive protein (CRP) level and alkaline phosphatase (ALP) level, were also reviewed. Additionally, some clinical parameters, such as age, sex, lesion location, tumour size on imaging, and tumour subtype, were adjusted and analysed.

### The definition of pathological complete response

After en block resection or amputation, pathologists evaluated the specimen and histopathologic information was obtained from the pathology report. The specimen was cut along the cross section with the largest area according to the preoperative imaging. The necrosis cell was observed by HE stain in tissue section. The necrosis rate was defined as necrotic area divided by total area. Patients whose rate were 90% or more had a pathological complete response (pCR) and patients whose rate less than 90% had a non-pCR.

### Regimens for NACT

All patients received first-line NACT, and the detailed regimens were AP: doxorubicin 45 mg/m^2^ + cisplatin 75 ~ 100 mg/m^2^ and MAP: high-dose methotrexate 8 ~ 12 g/m2 + doxorubicin 45 mg/m^2^ + cisplatin 75 ~ 100 mg/m^2^.

### Statistical analysis

The mean ± standard error and the median were used to present measurement data following a normal distribution and a skewed distribution, respectively. A receiver operating characteristic (ROC) curve and the Youden index (sensitivity + specificity-1) were used to determine the optimal cut-off values of the NLR due to the lack of a reference value in recent literature.

We used the chi-squared test and Fisher’s exact test to evaluate associations between the NLR, the PLR, other important clinical parameters, and pCR. Univariate and multivariate analyses by logistic regression models were conducted to confirm the independent factors that predict pCR. Statistical significance was considered when *p* < 0.05. All data were assembled using Excel 2007, and data analyses were performed by SPSS 22.0 software.

## Result

### Baseline data of the patients and tumours

A total of 403 patients histopathologically diagnosed with osteosarcoma of the extremities were identified. Ultimately, 96 patients met the inclusion/exclusion criteria and were included in this study. The baseline data are shown in Table 1. Among these 96 patients, 54 were male, and the median age at diagnosis was 17 years (range 7-45 years). Eighty-four (87.5%) tumours were located in the proximal tibia and distal femur, while 12 were located in other regions, including the proximal humerus, proximal fibula and distal tibia. The median tumour size measured on MRI was 195.37 ± 8.74 cm^3^. According to the Enneking surgical staging criteria, 28 patients were classified as stage I, 46 as stage II, and 22 as stage III.

**Table 1:** Association between clinical data and NLR/PLR

clinical parameters	n	NLR			PLR		
		LNLR (*n* = 50)	HNLR (*n* = 46)	*p*-value	LPLR (*n* = 30)	HPLR (*n* = 66)	*p*-value
Age (year)				0.447			0.823
≤ 17	56	31	25		18	38	
> 17	40	19	21		12	28	
Sex				0.959			0.165
Male	54	28	26		20	34	
Female	42	22	20		10	32	
Tumor location				0.949			0.363
Tibia	40	28	12		16	24	
Femur	44	30	14		10	34	
Others	12	8	4		4	8	
Tumor size (cm^3^)	195.37 ± 8.74	171.37 ± 12.94	218.34 ± 11.24	0.034	173.22 ± 10.54	201.97 ± 15.24	0.041
Enneking stage				0.024			0.321
I	28	18	10		10	18	
II	46	26	20		16	30	
III	22	6	16		4	18	
Subtype				0.202			0.362
Osteoblastic	58	34	24		15	43	
Chondroblastic	22	8	14		9	13	
Others	16	8	8		6	10	
ALP				0.013			0.763
Elevated	50	20	30		16	34	
Normal	46	30	16		14	32	
ESR				0.478			0.352
Elevated	70	38	32		20	50	
Normal	26	12	14		10	16	
CRP				0.148			0.161
Elevated	64	38	26		17	47	
Normal	32	14	18		13	19	
Mononuclear(10^9^/L)	0.64 ± 0.28	0.58 ± 0.23	0.66 ± 0.29	0.376	0.64 ± 0.20	0.65 ± 0.31	0.782

### The optimal cut-off values of the NLR and PLR

**Fig. 1:**
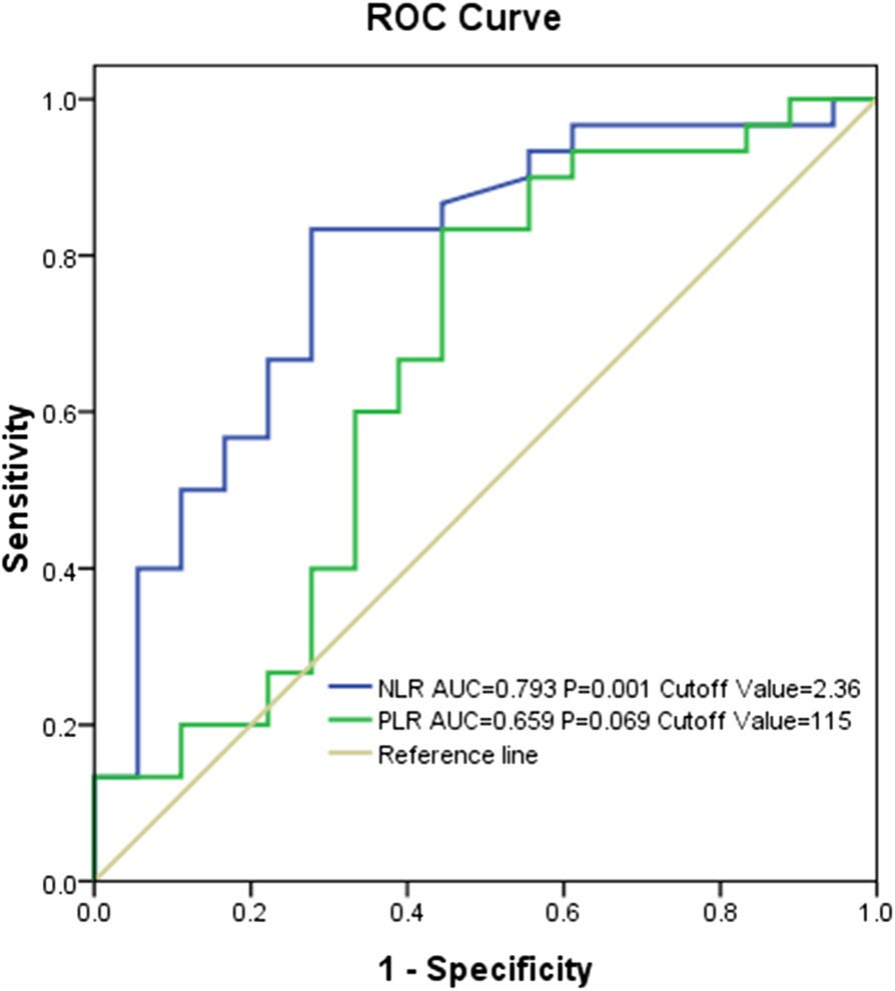
The areas under the ROC curves and the optimal cut-off values of neutrophil-to-lymphocyte ratio (NLR) and platelet-to-lymphocyte ratio (PLR)

As shown in Fig. 1, when pCR was defined as an end point, the areas under the curve (AUCs) for the NLR and PLR were 0.793 (*p* = 0.001) and 0.659 (*p* = 0.069), respectively. The optimal cut-off values of the NLR and PLR were 2.36 (sensitivity, 80.0%; specificity, 71.3%) and 115 (sensitivity, 83.3%; specificity, 55.6%), respectively. Subsequently, according to the optimal cut-off values of the NLR, patients were divided into two groups: the low NLR group (LNLR <2.36) with 50 patients and the high NLR group (HNLR ≥2.36) with 46 patients. Similarly, 66 patients were included in the low PLR group (LPLR <115), and 30 patients were included in the high PLR group (HPLR≥115).

### Associations of the NLR and PLR with clinical parameters

To investigate the relationships of the NLR and PLR with clinical parameters in osteosarcoma patients, comparisons between the LNLR (LPLR) and HNLR (HPLR) groups were conducted (Table 1). Our results demonstrated that the tumour size in patients with a low NLR was significantly smaller than that in patients with a high NLR (171.37 ± 12.94 cm^3^ vs. 218.34 ± 11.24 cm^3^, *p* = 0.034). A similar result was found in the PLR group (173.22 ± 10.54 cm^3^ vs. 201.97 ± 15.24 cm^3^, *p* = 0.041). Additionally, an advanced Enneking stage and elevated ALP were significantly associated with a high NLR, while the rates of these two parameters were not significantly different in the PLR group.

### Relationships between clinical parameters and pCR in univariate and multivariate analyses

Thirty-six patients (37.5%) achieved pCR after NACT for osteosarcoma. Univariate analysis was conducted to determine the risk factors that influenced pCR (Table 2). Our results revealed that patients with a smaller tumour volume, lower stage, lower NLR and lower PLR were more likely to achieve pCR. Subsequently, tumour size, Enneking stage, the NLR and the PLR were included in multivariate analyses. As shown in Table 3, the NLR before treatment was an independent risk factor for pCR. Compared to patients with a high NLR, those with a low NLR showed a more than 2-fold higher likelihood of achieving pCR (OR 2.82, 95% CI 1.36-5.17, *p* = 0.02).

**Table 2:** Association between patient/tumor data and pCR in univariate

Variable	n	pCR(%)	*P* value
Age (year)			0.669
≤17	56	22(39.3)	
>17	40	14(35.0)	
Sex			0.111
Male	54	24(44.4)	
Female	42	12(28.6)	
Tumor location			0.896
Tibia	40	16(40.0)	
Femur	44	16(36.4)	
Others	12	4(33.3)	
Tumor size (cm^3^)			0.034
≤ 195.37	38	20(52.6)	
> 195.37	58	18(31.0)	
Enneking stage			0.023
I	28	15(53.6)	
II	46	16(34.8)	
III	22	5(22.7)	
Subtype			0.263
Osteoblastic	58	23(39.7)	
Chondroblastic	22	6(27.3)	
Others	16	7(43.8)	
Regimens of NACT			0.261
AP	36	12(33.3)	
MAP	60	24(40.0)	
ALP			0.342
Elevated	50	21(42.0)	
Normal	46	15(32.6)	
ESR			0.406
Elevated	70	28(40.0)	
Normal	26	8(30.8)	
CRP			0.592
Elevated	64	25(39.1)	
Normal	32	11(34.4)	
NLR			0.021
LNLR	50	22(44.0)	
HNLR	46	14(30.4)	
PLR			0.031
LPLR	30	16(53.3)	
HPLR	66	20(30.3)

**Table 3:** Association between patient/tumor characteristics and pCR in multivariate analysis

Variable	OR	95%CI	*P* value
Tumor size≤195.37 vs >195.37	1.43	0.29-6.88	0.65
Enneking stage Ivs II/III	0.98	0.31-3.21	0.98
LNLR vs HNLR	2.82	1.36-5.17	0.02
LPLR vs HPLR	0.73	0.10-5.41	0.76

## Discussion

In this multicentre study, we explored the predictive value of the NLR for the effectiveness of NACT in patients with osteosarcoma of the extremities. Ninety-six patients undergoing NACT treatment were included, and the NLR and PLR were calculated. We found that a low pretreatment NLR (<2.36) was significantly associated with a higher rate of pCR and a better effect of NACT. However, other clinical parameters, including age, sex, tumour location, tumour size, clinical stage, tumour subtype, ALP, the ESR, CRP and the PLR, presented no relevance to pCR in our study.

Recently, many studies have demonstrated that the NLR plays a key role in predicting the treatment response to NACT and the survival rate in various cancers, such as colorectal cancer, rectal cancer, and breast cancer [17–21]. In a recent study by Chae and his colleague [22], the data of 87 patients with breast cancer were retrospectively analysed. They found that patients in the low NLR group had a higher rate of pCR than those in the high NLR group (42.1% vs. 18.4%, *p* = 0.018, 95% CI: 1.36–5.17). Kim et al. [8] used the pretreatment NLR and PLR as prognostic indicators of pCR in patients with gastric cancer. Kim IY et al. reported that an elevated NLR before NACT can be used as a predictor of a poor tumour response and an unfavourable prognosis in rectal cancer [19]. Our observations are consistent with those of previous studies.

Many oncologists and pathologists have suggested reasons for the association between a higher NLR in peripheral blood and tumour samples and higher-grade malignancy in cancer. Kk Liu et al. showed that an elevated NLR was significantly associated with higher pathological T stages and poor overall survival in bladder urothelial cancer cells [23]. The associations between an elevated NLR and an incomplete response and a poor prognosis in cancer are complex. We propose three main contributors to these associations. First, inflammatory cells in the tumour microenvironment can secrete a variety of cytokines, chemokines and cytotoxic mediators, which can induce early cell carcinogenesis and promote tumour occurrence [24]. Second, these cells can activate various downstream transcription factors, induce the expression of antiapoptotic genes and activate cyclin, thus promoting the survival and proliferation of tumour cells [25]. Moreover, inflammatory reactions can activate a variety of enzymes, which can increase the aggressiveness and metastatic potential of tumour cells by degrading extracellular matrix [26]. Neutrophils and lymphocytes are the most important cells in the inflammatory reaction and contribute to inflammation in the tumour microenvironment. Neutrophils can promote extracellular matrix reconstruction, tumour growth, metastasis and drug resistance [27–29] In addition, neutrophils promote angiogenesis by releasing vascular endothelial factors, including vascular endothelial growth factor (VEGF), thus promoting tumour invasion [30]. In contrast, lymphocyte-mediated cytotoxicity can inhibit tumour proliferation and metastasis [31]. In general, a higher NLR corresponds to a more severe systemic inflammatory response, suggesting that the tumour has greater invasiveness, higher malignancy and stronger drug resistance. Therefore, a higher NLR corresponds to a worse effect of NACT and a lower incidence of pCR.

## Limitation

Although this is multicenter study, the sample size is still relatively small. Meanwhile, this is a retrospective analysis, which may bring potential bias to the results. Thus, future multicenter prospective studies are needed to validate our findings.

## Conclusions

Our findings suggest that the NLR is an important factor predicting the response to NACT in extremity osteosarcoma patients. Patients with a higher NLR showed a lower percentage of pCR after NACT.

## Data Availability

All the data needed to achieve the conclusion are presented in the paper.

## Abbreviations

NACT: Neoadjuvant chemotherapy
NLR: Neutrophil-to-lymphocyte ratio
pCR: Pathological complete response
ROC: Receiver operating characteristic
SIR: Systemic inflammatory response
PLR: Platelet-to-lymphocyte ratio
WBC: White blood cell count
ESR: Erythrocyte sedimentation rate
CRP: C-reactive protein
ALP: Alkaline phosphatase
AUCs: Areas under the curve
VEGF: Vascular endothelial growth factor
